# Long-Range PCR and Nanopore Sequencing Enables High-Throughput Detection of *TCF4* Trinucleotide Repeat Expansions in Fuchs Endothelial Corneal Dystrophy

**DOI:** 10.1007/s40291-025-00803-8

**Published:** 2025-07-28

**Authors:** Bushra Alayed, Salina Siddiqui, Seema Anand, Chris F. Inglehearn, Christopher M. Watson, Manir Ali

**Affiliations:** 1 Division of Molecular Medicine, Leeds Institute of Medical Research, St James’s University Hospital, University of Leeds, Leeds, UK; 2 Department of Medical Laboratories, College of Applied Medical Sciences, Qassim University, Buraydah, Saudi Arabia; 3 The Eye Department, St James’s University Hospital, Leeds, UK; 4 North East and Yorkshire Genomic Laboratory Hub, Central Lab, St James’s University Hospital, Leeds Teaching Hospitals NHS Trust, Leeds, UK

## Abstract

**Introduction:**

Trinucleotide repeat expansion in CTG18.1, in intron 2 of *TCF4* (MIM *602272, #613267), is the main cause of Fuchs endothelial corneal dystrophy (FECD), accounting for around 75% of cases in Caucasians. CTG18.1 repeat expansion has typically been detected in peripheral blood genomic DNA by Southern blotting or short tandem repeat polymerase chain reaction (STR-PCR) combined with triplet-repeat primed PCR (TP-PCR) if needed. However both methods estimate the size of the expanded repeat relative to a size standard, and the former requires microgram amounts of DNA. To support the development of therapies, a high-throughput screening approach for repeat expansions in FECD is required. Here, we present a sensitive assay using long-range PCR and nanopore sequencing of genomic DNA to accurately resolve the CTG18.1 repeat.

**Methods:**

The CTG18.1 locus was analysed in genomic DNA from peripheral blood leukocytes by two different methods, and results were compared. The first approach used STR-PCR and capillary electrophoresis, followed by confirmatory testing of apparent homozygotes by TP-PCR. The second used long-range PCR, library preparation and long-read sequencing on an Oxford Nanopore Technologies MinION, with resolution of repeat length using the STRique algorithm.

**Results:**

CTG18.1 expansion was screened for in 119 patients with FECD and 83 controls, by STR/TP-PCR genotyping and, independently, by long-range PCR/long-read nanopore sequencing. Both methods gave comparable results, but the latter was also able to measure repeat length. A total of 73.1% of FECD cases (87/119) and 1.2% of age-matched controls (1/83) had at least one CTG18.1 expansion that was ≥ 50 repeats. The expanded CTG18.1 allele was inherited across multiple generations in four larger families, in a manner consistent with causing a dominant phenotype, revealing that some younger family members may be at risk. The G allele of SNP rs599550, ~1kb away from the expansion, is linked (in cis) with expanded alleles in 80.8% of FECD alleles with an expansion, compared with 12.5% in FECD alleles in cases without an expansion and 14.6% in Europeans.

**Discussion:**

We demonstrate that long-range PCR and long-read nanopore sequencing is a sensitive method requiring only nanograms of DNA, which can be scaled up for high-throughput detection and accurate sizing of CTG18.1 in peripheral blood DNA. The SNP, rs599550, is in linkage disequilibrium with the expansion and physically closer than rs613872, previously used in FECD association studies, making it better for use in diagnostic or association studies.

**Supplementary Information:**

The online version contains supplementary material available at 10.1007/s40291-025-00803-8.

## Key Points

**Table:** 

Long-range PCR and long-read nanopore sequencing can reliably detect and size expanded repeats in intron 2 of *TCF4* that cause FECD.
This new method requires nanograms of genomic DNA and can be scaled up with multiplexing to provide a high-throughput approach.
The SNP, rs599550, is physically closer to the expansion than rs613872, previously used in studies of FECD, and offers a new risk SNP for diagnostic and association study use.

## Introduction

Fuchs endothelial corneal dystrophy (FECD) is a bilateral, progressive eye disease that primarily affects the corneal endothelium and eventually leads to loss of vision. It affects approximately 1 in 14 individuals over 30 years old [1]. Expansion of a trinucleotide repeat, CTG18.1, in intron 2 of the *TCF4* gene, is strongly associated with FECD in Europeans, so much so that it appears to be in effect a near-Mendelian allele which is the main cause of the condition in this population, accounting for between 73 and 79% of cases [2–4]. This makes FECD the most prevalent repeat expansion disease in humans. There is gender bias, with three times more females affected than males [5], and it is the commonest cause of corneal transplantation worldwide [6].

The corneal endothelium is a monolayer of cells on the posterior surface of the cornea, in contact with the transparent water-like aqueous humour in the anterior chamber. It plays a vital role in maintaining the corneal stroma in a state of relative dehydration, which is essential for corneal clarity [7]. In FECD, gradual loss of endothelial cells on the posterior surface causes failure of the barrier function of the endothelium, leading to corneal oedema, clouding, glare and blurred vision. Dominant variants in *COL8A2* (MIM *120252, #136800), *ZEB1* (MIM *189909, #613270), *SLC4A11* (MIM *610206, #613268), *AGBL1* (MIM *615496, #615523) and *LOXHD1* (MIM *613072) have been reported to cause FECD but are much less common than the CTG18.1 trinucleotide repeat expansion in intron 2 of *TCF4*.

Comparing the size of the CTG18.1 repeat expansion in cultured corneal endothelial cells (CECs) with peripheral blood leukocytes from the same patient has revealed repeat expansions over ten times larger in size in the corneal cells [8, 9]. These larger expanded repeats cause disease by forming RNA nuclear foci that sequester RNA-binding proteins in corneal endothelial cells, leading to global mis-splicing with harmful consequences to the cell [10–12]. Hence, measurement of CTG18.1 alleles in peripheral blood leukocytes is only a marker of the somatic instability that causes much larger corneal expansions. CTG18.1 alleles measured in peripheral blood have been classified as expanded when they comprise more than 50 CTG trinucleotide repeats [2, 4]. Individuals harbouring at least one expanded allele have an approximately 76-fold increased risk of developing FECD [4].

Several different molecular techniques have been used to detect the CTG18.1 expanded repeat sequence in genomic DNA from blood. The choice of assay is dependent on the required sensitivity for detecting and sizing of the repeat and on the amount of DNA available. Southern blotting of restriction-digested genomic DNA is amplification-independent and can therefore be used to define the true upper size limit of the repeat. It has been used to detect expansions with over 1500 CTG repeats [2, 8] but is labour-intensive, low throughput and requires microgram amounts of genomic DNA. Polymerase chain reaction (PCR) with primers flanking the repeat region, followed by agarose gel or capillary electrophoresis, gives good sensitivity and specificity, requires small amounts of DNA and is scalable [2, 3]. However, this approach cannot accurately size expanded alleles of over 105 repeats [2]. A development of standard PCR which allows the detection of larger expansions, called triplet-repeat primed PCR (TP-PCR), adds a primer that binds within the repeat region, creating a ladder-effect pattern when visualised, though again the exact size of the repeat cannot be determined [3].

The repetitive nature of the CTG18.1 sequence and high GC content makes the expansion intractable to short-read next generation sequencing. Amplification-free, long-read sequencing using the RSII Pacific Biosciences instrument has been used on genomic DNA from blood leukocytes to provide a higher resolution analysis at the nucleotide level, though micrograms of genomic DNA were required, and only a limited number of samples were analysed [13]. More recently, Bionano optical genome mapping has been used to detect expansions encompassing 1800–11,900 repeats in length in genomic DNA from CECs [9].

Here we report the use of long-range PCR and long-read nanopore sequencing to detect and accurately size repeat expansions in blood leukocytes, and comparison of these results with the combined STR and TP-PCR approach. We find the long-range PCR/nanopore approach to be sensitive, cost-effective and scalable, providing a screening workflow that reliably detects and sizes CTG18.1 expanded repeat alleles in nanograms of genomic DNA from peripheral blood leukocytes, and we report its use in a cohort of patients with FECD from the north of England.

## Materials and Methods

### Patient Recruitment and Sample Collection

Patients with FECD and non-FECD controls were recruited in clinics in St James’s University Hospital Eye Department, Leeds, UK. Ethical approval was obtained from the Leeds East Research Ethics Committee (reference no. 17/YH/0032). Each individual’s age, gender and family history was documented. Patients with FECD were recruited in cornea clinics and were primarily of Northern European ancestry and over 50 years of age at recruitment, and 64.7% (77/119) were female. For multiple affected cases recruited from a known family, only one individual, chosen randomly, was included into the primary cohort to avoid bias. Non-FECD controls recruited from cataract clinics had their corneal endothelium inspected using a specular microscope prior to surgery, to exclude FECD. Genomic DNA was extracted from peripheral blood using either the QIAamp DNA Blood Midi Kit (Qiagen, Manchester, UK) or a Chemagic 360 automated nucleic acid extractor (Perkin Elmer, Waltham, MA, USA) according to the manufacturer’s instructions.

### Short Tandem Repeat (STR) and Triplet-Repeat Primed PCR (TP-PCR) Genotyping Assays

Initially, FECD cases and controls were subjected to STR genotyping performed on genomic DNA. Follow-up TP-PCR analysis was carried out on individuals in whom two alleles could not be identified using the STR assay. This either confirmed the presence of an expanded allele that could not be detected with STR analysis or verified the absence of an expansion indicating that the allele was homozygous for the size identified by STR. Oligonucleotide primer sequences were P1 (FAM-dAATCCAAACCGCCTTCCAAGT) and P2 (dCAAAACTTCCGAAAGCCATTTCT) for the STR assay, and primers P1, P3 (dTACGCATCCCAGTTTGAGACG) and P4 (dTACGCATCCCAGTTTGAGACGCAGCAGCAGCAGCAG) were used in the ratio of 1:1:0.3 for the TP-PCR assay. For the STR assay, 30 PCR cycles were carried out with a 94 °C denaturation step for 30 s, 61 °C annealing step for 30 s and 72 °C extension for 30 s, whereas for the TP-PCR assay, the conditions were 94 °C for 30 s, 60 °C for 45 s and 72 °C for 2 min for 40 cycles. PCR products were resolved on an ABI3130xl Genetic Analyser and analysed using GeneMapper (version 4.0) (Thermo Fischer Scientific, MA, USA).

### Long-Range PCR and Nanopore Sequencing

To perform target enrichment for long-read sequencing, the CTG18.1 locus was amplified by long-range PCR. Each 20 μL reaction consisted of 3 µL genomic DNA (~40 ng/µL), 10.24 μL of nuclease-free H_2_O, 2 μL of 10× SequalPrep reaction buffer (Thermo Fischer Scientific), 0.36 µL of 5U/µL SequalPrep long-polymerase, 0.4 µL of dimethyl sulfoxide, 2 μL of 10× SequalPrep Enhancer A and 1 µL each of 10 pmol/µL of forward (TNR1F-TAG: dTTTCTGTTGGTGCTGATATTGCCAACCCGTTTTCTTAACTAACAGC) and reverse (TNR1R-TAG: dACTTGCCTGTCGCTCTATCTTCTTACCAGTTTGATCGTCTCTTTGG) primers. Both primers contain universal tails (underlined) to enable a second round PCR incorporating a sample barcode. Thermocycling conditions comprised an initial denaturation step at 95 °C for 2 min, followed by ten cycles of 94 °C for 10 s, 57 °C for 30 s and 68 °C for 4 min. The next stage repeated the previous steps for a further 15 cycles but with the 4-min extension step further extended for 10 s after each cycle up to 6.5 min. This was followed by a final 5-min extension step at 72 °C. Once the PCR cycles completed, an aliquot of the reaction was resolved by electrophoresis on a 1% agarose gel to verify successful amplification.

**Fig. 1:**
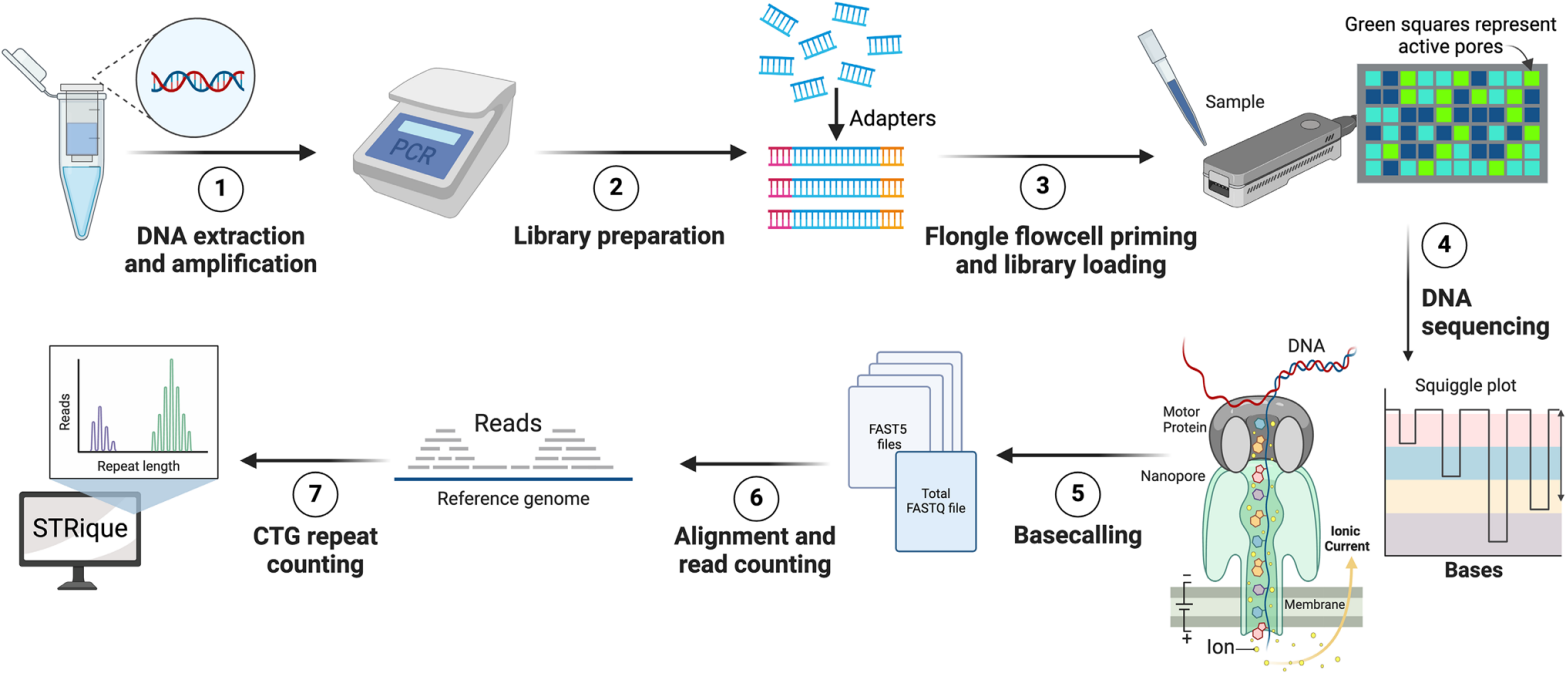
Long-read nanopore sequencing workflow. (1) Long-range PCR was performed to amplify the CTG18.1 repeat and flanking sequences; a second-round PCR incorporated a unique barcoded index for each sample. (2) Samples were combined in equimolar quantities and a sequencer-compatible library was prepared from the pool of indexed amplification products; end-repair and adapter ligation reactions were completed. (3) Flongle flowcells were primed, and the prepared library was loaded. (4) Nanopore sequencing was initiated with motor proteins that facilitate the movement of unwound DNA strands into the nanopores. As DNA passes through the pores, the disrupted flow of hydrogen ions creates characteristic “squiggle plots” that are captured for post-run processing. (5) Raw data (FAST5 files) were demultiplexed using the per-sample barcode then base-called and converted to FASTQ format. (6) Reads were filtered according to their size and quality then aligned to the human reference genome (build hg19) to maintain consistency with downstream use of the data in line with a previous study [16]. (7) STRique was used to count the number of CTG18.1 repeats. Created in BioRender. Alayed, B. (2025) https://BioRender.com/j41p831

The nanopore sequencing workflow is illustrated in Fig. 1. Amplification products were first purified from the rest of the reaction components using AppMag PCR clean-up beads (Appleton Scientific, Birmingham, UK). To multiplex multiple samples in a single sequencing run, amplification products from each pre-indexed PCR served as a template for a second round of PCR, incorporating a unique barcode from the Barcoding Expansion Pack (EXP-PBC096; Oxford Nanopore Technologies, Oxford, UK). Each 50 μL reaction comprised 25 µL of 2× LongAmp Taq master mix (New England Biolabs, Hitchin, UK), 1 μL of a unique PCR barcode primer (one of BC1-BC96 at 10μM) and 24 μL of purified tailed PCR template containing between 100 and 200 fmol DNA diluted in nuclease-free water. Thermocycling conditions for the second round PCR were 95 °C for 3 min, followed by eight cycles of 95 °C for 15 s, 62°C for 15 s and 65°C for 4 min, then a final 4-min extension step at 65 °C. An aliquot of each PCR was verified for size by agarose gel electrophoresis. Equimolar pools of ten barcoded samples (50 ng per library) were combined for each Flongle run (FLO-FLG001, R9.4.1; Oxford Nanopore Technologies), while for a MinION run (FLO-MIN106D; Oxford Nanopore Technologies), 45 independent samples of equal amounts (222 ng each) were pooled into 10 μg, from which 1 μg was used for MinION library preparation. To prepare the library, the amplified products in the pooled sample were end-repaired using the NEBNext Companion Module (New England Biolabs), cleaned up using AppMag beads (Appleton Scientific) and eluted with 61 μL nuclease-free water. Adaptors were ligated to the DNA ends using the Ligation Sequencing Kit (SQK-LSK110; Oxford Nanopore Technologies), purified using AppMag beads (Appleton Scientific) which were washed twice with 250 μL Long Fragment Buffer (Oxford Nanopore Technologies) and eluted with 15 μL of Elution Buffer (Oxford Nanopore Technologies). The library was quantified using a Qubit Broad Range assay kit (Thermo Fischer Scientific), and molarity was calculated with NEBioCalculator (https://nebiocalculator.neb.com/#!/ligation). For the Flongle flowcell, 3–20 fmol of the library was combined with 15 μL of sequencing buffer and 10 μL of loading beads. For the MinION flowcell, 50 fmol of pooled library, made up to 12 μL with nuclease-free water, was added to 37.5 μL of sequencing buffer and 25.5 μL of loading beads, subsequently loaded onto the flowcell according to the manufacturer’s instructions (https://nanoporetech.com/). All the data were collected using the same version of MinKNOW (22.10.10).

### Bioinformatics Analysis

Raw data generated in FAST5 format was demultiplexed using the unique per-sample barcode and base-called with Dorado (version 0.5.2; https://github.com/nanoporetech/dorado). NanoStat (version 1.6.0; https://github.com/wdecoster/nanostat) was used to assess read length, quality and the total number of reads generated [14]. Reads were filtered to retain those that were 4000–6000 bases in length and had a quality value > Q10 using NanoFilt (version 2.8.0; https://github.com/wdecoster/nanofilt). Reads were next aligned to the human reference genome (hg19) using Minimap2 (version 2.26; https://github.com/lh3/minimap2) [15] to generate a Sequence Alignment Map (SAM) file. This was sorted by genomic coordinate and converted to Binary Alignment Map (BAM) format using Samtools (version 1.19; https://github.com/samtools/samtools).

To size the CTG18.1 repeats, demultiplexed FAST5 files together with the SAM file were analysed with STRique (version 0.4.2) (Short Tandem Repeat identification, quantification and evaluation; https://github.com/giesselmann/STRique) [16]. STRique located the boundaries of the CTG18.1 repeat (chr18:53,253,387-53,253,458; build hg19) and quantified the repeat counts for each read. Results were reported in .tsv format and reviewed using Microsoft Excel. The CountIf function identified the frequency of each repeat size, and data were reported as a histogram. Repeat counts below 5, which were present in all the samples tested, were considered artefacts following conventional Sanger sequencing (Supplementary Fig. 1). The most frequent repeat size around a normal distribution was assigned the value for the repeat. For increased confidence in sizing repeats, only values with read depths greater than 10 were considered.

Sequence variants were identified from the BAM files using Clair3 (version 1.0.5) [17] which generated data in variant call format. Aligned sequence reads were visualised using Integrative Genomics Viewer (version 2.16.2) (IGV; https://www.broadinstitute.org/igv/). When two alleles were similar in size, these variants were used to determine phase to distinguish between reads from each allele, and thus provide accurate sizes for each expansion.

### Sanger Sequencing

Sanger sequencing was carried out on an aliquot of the first round long-range PCR product that had been treated with ExoSAP-IT (Thermo Fischer Scientific), using either TNR2F (dCCCTAATTGGTTTCCCTCTTCTTC) or TNR2R (dCATCCCTTTGCTTCCTTTTCCTAA) nested primers and the BigDye Terminator version 3.1 Cycle Sequencing kit (Thermo Fischer Scientific). Sequencing reactions were resolved on an ABI3130xl Genetic Analyzer according to the manufacturer’s instructions (Thermo Fischer Scientific), and chromatograms were visualised using Sequence Analysis (version 5.2) (Thermo Fischer Scientific).

### Statistical Analysis

For the CTG18.1 repeat, an allele was classified as expanded if it had ≥ 50 copies of the repeat. Comparison between cases and controls was performed in a 2 × 2 contingency table using a Fischer’s exact test calculation at the 1% level of significance.

## Results

### Comparison of STR/TP-PCR Genotyping and Long-Read Nanopore Sequencing

Standard STR and TP-PCR genotyping was performed on genomic DNA from 109 of the 119 patients with FECD recruited (Supplementary Table 1). Although this approach supported identification of the cases with an expansion, the largest repeat size that could be assigned was 35 repeats. Wieben and colleagues [2] reported expanded alleles of up to 105 repeats using the STR assay, which is likely to result from the use of a 3-min PCR extension step, as opposed to the 30-s extensions used in the study described here.

**Fig. 2:**
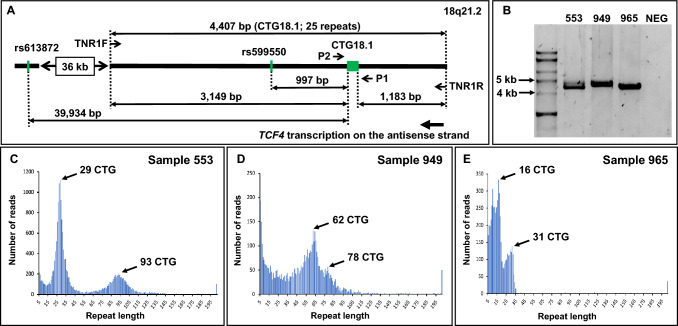
Long-range PCR and nanopore sequencing. **A** Schematic overview of the CTG18.1 locus within the *TCF4* gene cytoband 18q21.2. The trinucleotide repeat sequence (CTG18.1) and SNPs (rs599550 and rs613872) are displayed in green with nucleotide base pair (bp) distances between each feature. For nanopore sequencing, oligonucleotide primers TNR1F and TNR1R amplify the CTG18.1 locus, generating a 4407-bp PCR fragment that contains 25 repeats. For STR genotyping, primers P1 and P2 amplify a 186-bp fragment. **B** Gel image showing PCR products > 4400 bp following TNR1F and TNR1R amplification from patients 553, 949 and 965 respectively. A no-DNA control lane is indicated (NEG). Histograms generated from STRique output for patients 553 (**C**), 949 (**D**) and 965 (**E**) are shown. The *x*-axis defines the repeat size interval and *y*-axis defines the number of reads containing a repeat in the specified range. Reads with greater than 50 CTG repeats are considered an expanded allele. Note, sample 965 is biallelic for two normal-sized alleles of 16 and 35 repeats; sample 553 has a normal-sized allele of 29 repeats and an expanded allele of 93 repeats; sample 949 has two expanded alleles of 62 and 78 repeats

To overcome this limitation in the sizing of expanded repeats, long-range PCR spanning the CTG18.1 locus was performed in combination with long-read nanopore sequencing, as described in Materials and Methods. Data were generated for 119 locally recruited patients with FECD, including the 109 previously genotyped by STR/TP-PCR. Results with each method were very similar, with only small size differences noted, confirming that these methods consistently replicate findings, including distinguishing between heterozygous and homozygous repeat expansion cases (Supplementary Table 1). Should the expanded alleles be very similar in size, wherever possible, variants in the target region of the nanopore sequence were used to phase the two alleles, to provide an accurate measurement for the repeat sequence. All expanded alleles in the FECD cohort could be sized following nanopore sequencing by STRique analysis, whereas before, they could only be classed as either expansion positive or expansion negative. To assess sequence quality, base calling metrics were analysed across the targeted region for seven flongle runs (Supplementary Table 2). Results of long-range PCR/nanopore sequencing are shown for three representative FECD cases in Fig. 2.

### Comparison of Repeat Sizes between Patients with FECD and Controls

**Fig. 3:**
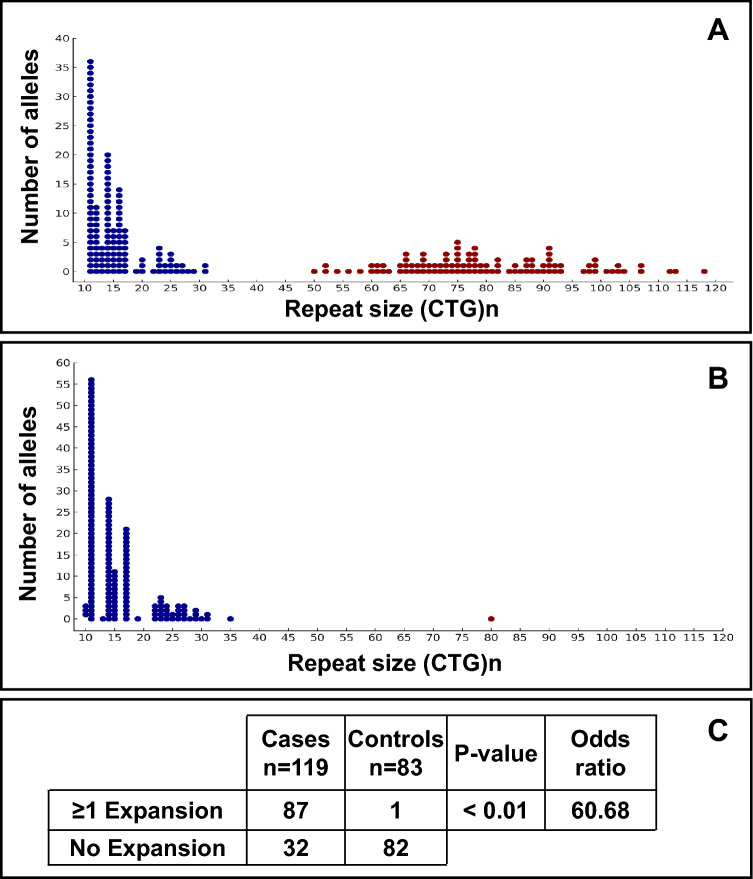
Comparison of CTG18.1 repeat length between patients with FECD and controls. Dot plot showing repeat size (CTG)*n*, where *n* is the number of repeats represented on the *x*-axis and number of alleles of that size on the *y*-axis for FECD cases (**A**) and controls (**B**). Blue dots represent the normal allele range with fewer than 50 CTG repeats, whereas red dots signify a mutant allele size expansion of greater than or equal to 50 repeats. **C** Table showing number of cases versus controls with one or more expansion and without an expansion. The observation that FECD cases were more likely to have an expanded CTG18.1 allele was statistically significant (*p* < 0.01), with an odds ratio of 60.68

Nanopore sequencing of the CTG18.1 expansion in 119 FECD cases revealed that 87 (73.1%) harboured at least one (*n* = 75) and in some cases two (*n* = 12) expansions of ≥ 50 CTG repeats that were likely to cause or contribute significantly to their disease phenotype (Fig. 3A). This analysis was also able to identify and size the CTG18.1 locus in the second, non-expanded allele of cases heterozygous for a repeat expansion. In addition, genomic DNA from 83 non-FECD controls was genotyped using the STR and TP-PCR assays (Supplementary Table 3). Biallelic variants less than 35 repeats accounted for almost all of the control genotypes (Supplementary Table 3 and Fig. 3B). Four control samples, which required further clarification following TP-PCR, were also nanopore sequenced. One of these was confirmed to be heterozygous for an expanded allele (80 repeats), while the remaining three proved expansion negative. No CTG18.1 alleles in the intermediate size range (35–49 repeats) were identified in either cases or controls. These observations strongly support previous reports that alleles with more than 50 repeats are significantly associated with FECD (*p* < 0.01) (Fig. 3C) and suggest that individuals carrying an expanded CTG18.1 allele have a 60-fold increased risk of developing FECD.

### Familial Transmission of Repeat Expansions at the CTG18.1 Locus

**Fig. 4:**
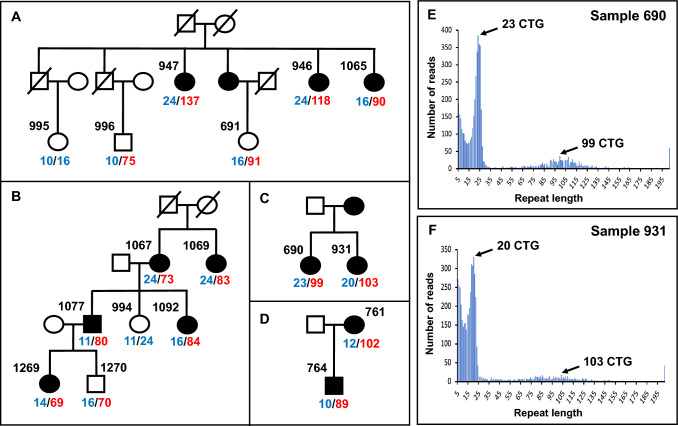
Familial transmission at the CTG18.1 locus. Pedigrees for FECD cases with multiple affected members. Families FECD 1–4 are shown (**A–D**). Patient IDs are indicated for individuals for whom genomic DNA was available for nanopore sequencing. Values indicated in blue define non-expanded repeats, whereas alleles in red define expansion of greater than 50 repeats. Note, autosomal dominant transmission of the expansion with the disease phenotype (**B–D**) and asymptomatic cases (**A**; 691, 996 and **B**; 1270) that harbour an expansion. Histogram plots generated from the STRique output for patients 690 (**E**) and 931 (**F**) are shown. Reads with greater than 50 CTG repeats are considered an expanded allele. Note the presence of broad, uneven peaks making it harder to determine a single repeat size

Four families with multiple affected cases were also genotyped for the CTG18.1 repeat expansion by long-range PCR and nanopore sequencing (Fig. 4A–D) to determine the extent to which expanded alleles stably transmit through meiosis. The expansion allele was transmitted consistently from one generation to the next and could be seen to segregate with FECD. However, it was observed that the size of the expanded repeat varied between individuals within the same family, even within the same generation. This seems likely to reflect instability of the expanded repeat at meiosis. Indeed, some cases were observed with expansion alleles that were not normally distributed but instead had broad or several uneven peaks, suggesting that some variation in repeat size in blood leukocytes from patients was also somatic in origin (Fig. 4). Such somatic mosaicism made it harder to determine a single repeat length for the larger expansions and likely contributed to the increased variability in estimates of repeat expansion size in the families. Inter-generational differences appeared independent of the affected parent’s gender. At the time of testing, three asymptomatic individuals in these pedigrees were found to have inherited an expanded allele, yet presented no symptoms. All three were under the age of 50 years, implying that they were ‘at risk’ but had not yet progressed to FECD because of their younger age.

### Haplotyping at the CTG18.1 Locus

One additional benefit of single molecule long-read nanopore sequencing over traditional short-read sequencing methods is the ability to define variants that are in *cis* (linkage disequilibrium) with the CTG18.1 repeat. The common single nucleotide variant rs599550 (chr18:55,585,157 (GRCh38); Ref nucleotide: G, Non-ref nucleotide: A) is located 997 base pairs (bp) proximal (centromeric) to the CTG18.1 trinucleotide repeat sequence (Fig. 1A). The reference-matching G allele of rs599550 has a frequency of 0.146 in European (non-Finnish) alleles in gnomAD (version 4.1.0) (Table 1). Haplotypes from FECD cases were assigned to one of three groups on the basis of presence or absence of a repeat expansion: expansion carrying; non-expansion carrying second strands from heterozygous FECD cases; and non-expanded haplotypes from cases with no expansion. Of the 119 FECD cases, 75 individuals had a monoallelic CTG18.1 expansion ≥ 50 CTG repeats, and 12 individuals had a biallelic expansion, giving a total of 99 CTG18.1 expansion-positive haplotypes. The G allele of rs599550 was present on 80.8% of these, showing significant enrichment of the G allele over that seen in gnomAD. In contrast, frequency of the rs599550 G allele in the 75 haplotypes of the second non-expanded strands in expansion heterozygotes was 8.0%, and frequency in the 64 haplotypes found in 32 non-expansion carrying FECD cases was 12.5%, neither of which differ significantly from the population frequency (Table 1). This over-representation of the G allele of rs599550 on the same haplotype with the expanded repeat suggests the possibility of a common founder allele in the European FECD population.

**Table 1:** Analysis of linkage between the CTG18.1 repeat and SNP rs599550

	Number of alleles	rs599550		*p* value
		A	G	
gnomAD	501,210	428,154 (0.8542)	73,056 (0.1458)	
EXP-POS (≥ 50)	99	19 (0.1919)	80 (0.8081)	< 0.01
EXP-POS (< 50)	75	69 (0.9200)	6 (0.0800)	NS
EXP-NEG (< 50)	64	56 (0.8750)	8 (0.1250)	NS

Frequency of single nucleotide variant, rs599550, A or G nucleotide amongst the gnomAD alleles in the European (non-Finnish) population; the expanded alleles in the FECD cohort (EXP-POS (≥ 50)); the non-expanded alleles in cases with a monoallelic expansion (EXP-POS (< 50)); and the non-expanded alleles in FECD cases that did not have an expansion (EXP-NEG (< 50)). Statistical significance was indicated at the 1% level. Note that linkage of the CTG18.1 repeat expansion with the G allele of rs599550 was statistically significant (*p* < 0.01)

*NS* not significant

## Discussion

Here we demonstrate that long-range PCR combined with long-read nanopore sequencing is a reliable method for detecting the size of the CTG18.1 triplet repeat sequence in *TCF4* in genomic DNA extracted from peripheral blood. The results obtained consistently replicate those obtained using the STR/TP-PCR approach used in this study, with only small differences in the sizes obtained. Comparison of sizing for expanded alleles with more than 35 repeats was not possible because the STR assay was performed with a PCR extension step of 30 s, as opposed to 3 min, an approach which has been used by others [2] to size up to 105 repeats. Nanopore sizing measures sequence directly and so is more accurate at sizing the repeat expansion, compared with STR/TP-PCR, which relies on fragment size estimation relative to a size standard. In contrast to amplification-free methods such as Southern blotting [2] or CRISPR-Cas9 target enrichment and Pacific Biosciences sequencing [13], very little starting DNA is required (nanograms compared with micrograms of genomic DNA). Nanopore sequencing also allows the pooling of libraries prepared from multiple different individuals, each labelled with a unique barcode, to create a high-throughput, multiplexed workflow. The cost of making libraries and running the pooled sample on a Flongle flowcell was approximately £30 per sample in a pool of 10, and varied from £26 to £18 per sample depending on the number of pooled samples from 45 to 90, when ran on the MinION device. While this study was conducted using a workstation-attached standalone Flongle or MinION flowcell instrument, the widespread availability and adoption of the GridION (Oxford Nanopore Technologies), with integrated compute, would ensure a controlled software environment and streamline diagnostic pipeline for implementation in standard clinical laboratories.

The use of nanopore sequencing to study the CTG18.1 repeat sequence in genomic DNA from patients with FECD has also been described recently by another group [18]. However, they used a standard, short-range PCR approach for target enrichment, as opposed to long-range PCR used in our study. This appeared to work in their cohort of 92 FECD cases from Turkey, though they did not find any expansions over 40 repeats in their patients. This may be due to ethnic differences in the causes of FECD, with the repeat expansion being very common (> 70%) in our Caucasian cohort. However the absence of any patients with a repeat expansion seems unusual and suggests a problem in the methods used in their study. Unlike the study presented here, they did not verify their results using two different methods.

Although there are other data analysis programs available for the analysis of triplet repeat expansions such as RepeatHMM [19] and Repeat Detector [20], we chose STRique [16] to measure the size of the CTG18.1 repeat sequences in *TCF4*. STRique had previously been used to study the hexanucleotide repeat expansion within *C9ORF72,* which causes frontotemporal dementia and amyotrophic lateral sclerosis (MIM #105550) [16]. These authors used amplification-free, CRISPR-Cas9-based enrichment of the repeat sequence, whereas this study used long-range PCR to exponentially amplify the target sequence, and consequently, quantification using STRique of large amounts of PCR product proved computationally demanding. Nevertheless, we were able to determine the expansion in all local FECD cases tested using this software.

The largest CTG18.1 repeat detected in peripheral blood genomic DNA from patients with FECD in this study was 137 repeats (Family FECD-1, case 947). The upper limit of the long-range PCR/nanopore sequencing assay has yet to be determined, though bioinformatics analysis to retain sequence reads up to 6000 bp sets a theoretical limit of approximately 500 trinucleotide repeats as the upper boundary for this assay. Recent studies have shown that the expanded repeat observed in genomic DNA from CECs is over ten times larger than that detected in blood leukocytes from the same patient, revealing somatic instability of the repeat in different tissues [8, 9]. This suggests that the cause of FECD may be a very large expansion of the CTG18.1 repeat which arose somatically in CECs, for which the much smaller expansion in genomic DNA from blood is only a marker. These tissue-specific expansion differences in somatic cells may be due to a combination of genetic, epigenetic, transcriptional, cellular and environmental factors. Each tissue has its own unique set of characteristics, including replication mechanisms, DNA repair pathways, chromatin structure, gene expression profiles and exposure to stress, all of which can impact the stability of repetitive DNA sequences. Understanding these mechanisms will help to unravel the pathogenesis of these diseases and may provide insights for potential targets to stabilise repeat regions and mitigate disease progression in the future. It would be interesting to test the long-range PCR/nanopore sequencing approach on larger repeat expansions observed in CECs and to determine whether repeat size varies over time. However, the inaccessibility of CECs, by contrast with the ready accessibility of genomic DNA from blood, means that any diagnostic or longitudinal testing is likely to focus on genomic DNA from peripheral blood leukocytes for the foreseeable future.

Previous studies have considered the threshold for CTG18.1 repeat size as pathogenic when more than 40 [3, 21, 22] or 50 repeats [2, 4] are detectable in peripheral blood leukocytes. In this study, all expansion alleles identified in patients with FECD had ≥ 50 repeats. In this cohort of patients with FECD from the north of England, almost entirely of European ethnicity, 73.1% (87/119) had at least one expansion repeat allele, which is consistent with previous reports in northern European patients with FECD [2–4, 21]. Epidemiological studies of CTG18.1 alleles in patients with FECD from other populations have identified the expanded repeat in 51.3% (97/189) of Australian [23], 43.8% (25/57) of Chinese [24], 38.9% (21/54) of Thai [25], 35.0% (21/60) of African American [22], 34.1% (15/44) of Indian [26] and 25.5% (12/47) of Japanese [27] cases. All of these studies showed significant enrichment of the repeat expansion in cases over controls. Our own study, using controls which had been examined clinically and confirmed to be disease negative, identified only one expansion allele in 83 individuals. Other studies in Europeans using clinically examined, age-related subjects without any signs of FECD [2, 3, 21], or a cohort of patients with age-related macular degeneration as controls [4], found that only between 3% and 7% of control subjects carried the CTG18.1 expanded allele. These findings confirm that expansion of the CTG18.1 repeat greatly increases risk of FECD in older age, in a manner consistent with it acting as a near fully penetrant dominant Mendelian allele.

This study also reports parent to child transmission of FECD in four unrelated families, with co-segregation of an expanded CTG18.1 repeat in blood leukocyte genomic DNA, again consistent with the repeat expansion acting as a dominantly inherited allele of near-complete penetrance in those over 50 years of age. Similar findings were reported previously [3]. Here we noted small variations in size of the expanded repeat between individuals within the same family, implying meiotic instability. Meiotic instability of repeat expansions prior to germline transmission could be caused by replication slippage, mismatch repair mechanisms, and repeat regions flanked by an open chromatin structure that allow easier access for replication and repair machinery, further increasing repeat instability. Gender of the transmitting parent can also influence repeat instability in repeat expansion disorders, due to differences in the replication and repair processes during gametogenesis. For FECD, there appears to be no obvious gender bias in intergenerational transmission, on the basis of this and the previous reports [3, 28], though the numbers examined are small. In the study reported here and the previous study [3], asymptomatic individuals in the younger generations were found to carry the expanded CTG18.1 allele. Two families (FECD 1 and FECD 2) with three such asymptomatic individuals (samples 691, 996 and 1270) were identified in this study. This is most likely due to the younger age of these individuals, meaning they are at risk of developing FECD in the future. Pre-symptomatic diagnosis of cases may prove useful, since there is ongoing interest in developing treatment strategies for targeting the repeat expansion in FECD [4, 29, 30].

Previous studies have highlighted association of the G allele of rs613872, located approximately 40 kb from the CTG18.1 expansion, with FECD [3, 31]. They documented that the G allele, which is present in 12% (*n* = 200) and 15% (*n* = 820) of Caucasian control alleles, accounted for 46.7% (*n* = 240) and 40% (*n* = 560) of all alleles in their cases. Here we sought to investigate whether any SNPs in sequence covered by the long-range PCR adjacent to the CTG18.1 repeat were also informative and might therefore prove useful in association studies or diagnostic screening. We found that the G allele of rs599550, less than 1 kb away from CTG18.1 and genotyped in the assay described here, accounts for 14.6% of alleles in the European population (*n* = 501,210) and is present in 39.5% (*n* = 238) of the FECD alleles that were studied in this report. However, we also demonstrate directly, for the first time, that the G allele of rs599550 is present on 80.8% of the *TCF4* expansion-containing haplotypes. This highly significant enrichment in cases suggests that this SNP may be a better marker for repeat expansion than rs613872 as it is closer to the repeat. Furthermore, the strong association seen between this SNP and CTG18.1 repeat expansion suggests that repeat instability causing FECD may have originated in a common founder allele within this population, though further studies are required to clarify the relevance of this observation. The G allele of rs599550, accounting for 0.05% of East Asian (*n* = 41,120), 2.54% of African/African American (*n* = 60,066) and 15.29% of South Asian (*n* = 73,570) alleles, could also be used to extend association FECD studies beyond European patients. SNP rs599550 has been studied as a screening biomarker in genome-wide association studies looking at common diseases and traits in the UK Biobank. The A allele of rs599550 was one of five SNPs significantly associated with increased social isolation after the coronavirus disease 2019 (COVID-19) pandemic, and these same SNPs were used in a Mendelian randomisation study to highlight a causal link with increased risk of osteoarthritis [32].

The expanded repeat in *TCF4* is thought to cause CEC death by RNA toxicity that results from forming nuclear RNA foci which sequester RNA-binding proteins and cause deleterious events due to abnormal splicing [10–12]. Another mechanism of FECD onset in cases lacking a CTG18.1 repeat expansion may be dysregulation of specific *TCF4* transcript isoforms [33]. Multiple different *TCF4* transcript isoforms exist as a result of differential 5′-exon usage and alternative splicing [34, 35]. Following total transcriptome analysis of *TCF4* between expansion-positive and expansion-negative CECs, a skewing of the ratios of the transcript isoforms was observed [31]. That study also identified rare, potentially deleterious single nucleotide variants in *TCF4* in 7 of 134 expansion-negative cases, which the authors suggested may be a risk factor for this transcript dysregulation. It is possible that some of the 32 CTG18.1 expansion-negative cases in the Leeds FECD cohort may also carry a rare, regulatory *TCF4* variant, though variants in other genes implicated in FECD, including *COL8A2* [36], *SLC4A11* [37], *ZEB1* [38], *AGBL1* [39] or *LOXHD1* [40], could also account for their condition. Whole exome or genome sequencing will provide further insights into the cause of disease in these expansion-negative cases.

This study has a number of limitations. The assay described is amplification-based, which could be prone to PCR bias whereby there is preferential amplification of shorter-sized alleles over larger expanded alleles. This would tend to under-represent extremely large expansions in the analysis. The assay successfully identified an allele of 137 repeats, but the upper limit of amplification was not determined. Furthermore, to date, the assay has only been tested on genomic DNA from peripheral blood, which is easily biopsied, rather than cornea where pathology manifests. When comparing allele sizes between the long-range PCR/nanopore sequencing method and the STR assay, a 30-s rather than a 3-min PCR extension step was used in the latter, precluding the accurate sizing of alleles with more than 35 repeats. In addition, the study only analysed a modest-sized cohort of European patients, and only four relatively small families were recruited, so findings apply only to patients with FECD of similar ethnic origin and would benefit from replication in other/larger cohorts.

Nevertheless, here we have shown that long-range PCR combined with long-read nanopore sequencing can detect and size the trinucleotide repeat expansion in *TCF4* that is the likely cause in over 70% of FECD cases in Caucasians. The method uses nanograms of genomic DNA and can be scaled for high-throughput screening. The expanded allele is transmitted with FECD in families in a manner consistent with dominant inheritance and can be detected in younger generations before any sign of disease onset, providing a potential early diagnostic screen for FECD. The assay also generates a haplotype which includes SNP rs599550, less than 1kb from the repeat, with the relatively rare (14.6%) G allele greatly enriched in the expansion-carrying haplotype. The approach described here could be used in diagnostic screening in at-risk cases and could also prove useful for stratifying patients for future precision therapies currently being developed.

## Supplementary Information

Below is the link to the electronic supplementary material.

Supplementary file1 (PDF 608 kb)

## Declarations

### Data availability

All the data supporting the findings of this study are available within the paper and Supplementary Information.
