# Supplementary material for: Long-Range PCR and Nanopore Sequencing Enables High-Throughput Detection of TCF4 Trinucleotide Repeat Expansions in Fuchs Endothelial Corneal Dystrophy
Source: Mol Diagn Ther. 2025 Jul 28;29(6):801–12. doi: 10.1007/s40291-025-00803-8 (PMC12578760; doi:10.1007/s40291-025-00803-8)
Supplement: Supplementary file 1 — Supplementary file1 (PDF 608 kb) [file 40291_2025_803_MOESM1_ESM.pdf]

Supplementary information

Long-Range PCR and Nanopore Sequencing Enables High Throughput Detection of *TCF4* Trinucleotide Repeat Expansions in Fuchs Endothelial Corneal Dystrophy.

Authors and Affiliations

Bushra Alayed[1,2], Salina Siddiqui[1,3], Seema Anand[3], Chris F. Inglehearn[1], Christopher M. Watson[1,4], Manir Ali[1]

1: Division of Molecular Medicine, Leeds Institute of Medical Research, St. James's University Hospital, University of Leeds, Leeds, UK.

2: Department of Medical Laboratories, College of Applied Medical Sciences, Qassim University, Buraydah, Saudi Arabia.

3: The Eye Department, St. James's University Hospital, Leeds, UK.

4: North East and Yorkshire Genomic Laboratory Hub, Central Lab, St James's University Hospital, Leeds Teaching Hospitals NHS Trust, Leeds, UK.

Correspondence to Manir Ali. m.ali@leeds.ac.uk

ORCID number:

Bushra Alayed: umbmaa@leeds.ac.uk
Salina Siddiqui: salinasiddiqui@nhs.net
Seema Anand: seema.anand3@nhs.net
Chris F. Inglehearn: 0000-0002-5143-2562; c.inglehearn@leeds.ac.uk
Christopher M. Watson: 0000-0003-2371-1844; c.m.watson@leeds.ac.uk
Manir Ali: 0000-0003-3204-3788; m.ali@leeds.ac.uk

Running Title: Nanopore sequencing detects *TCF4* trinucleotide repeat expansions in Fuchs corneal dystrophy.

Supplementary Figure 1: Comparison of nanopore sequencing/STRique output against Sanger sequencing.

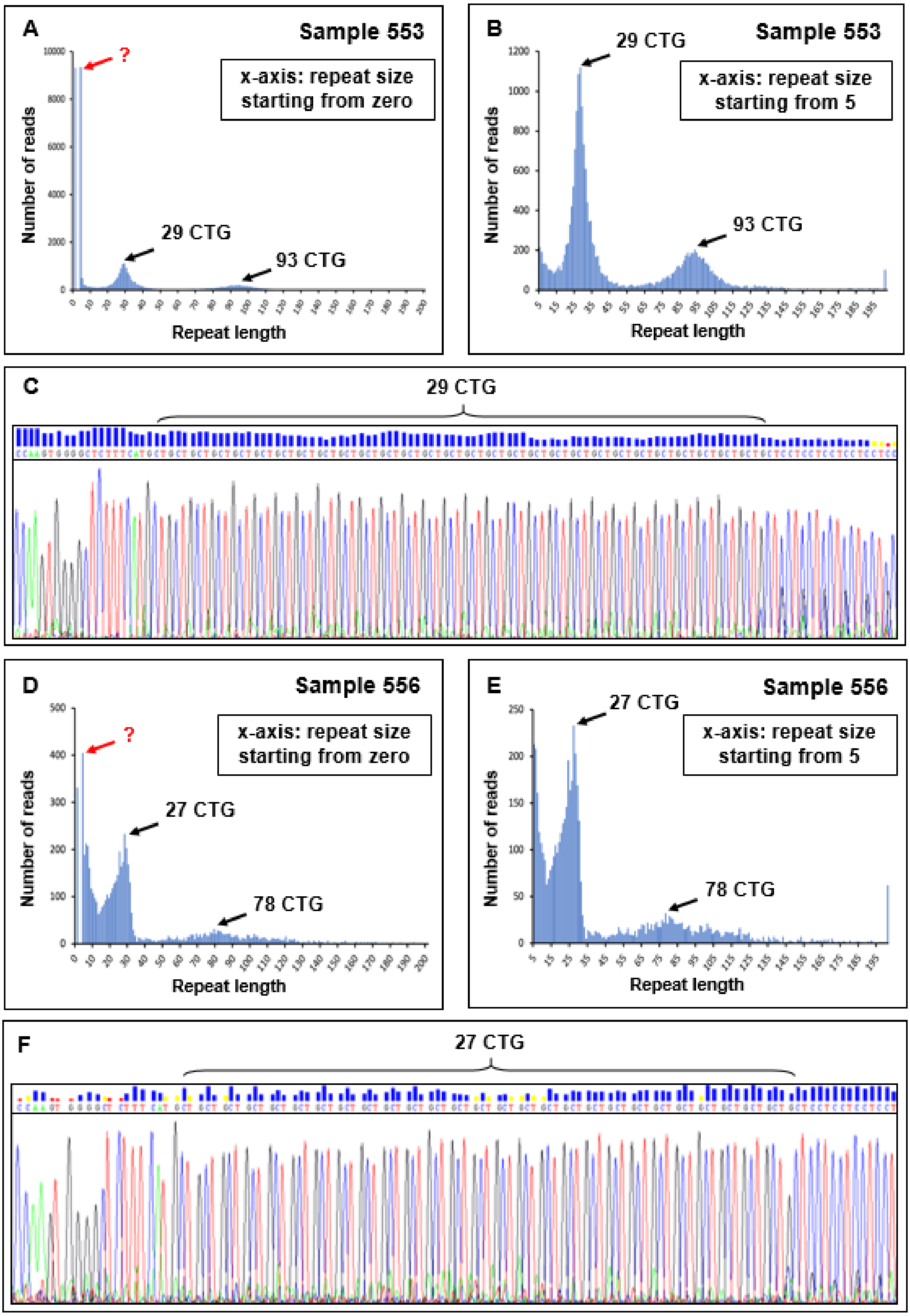

Histograms generated from STRique output following nanopore sequencing for patients 553 **(A & B)** and 556 **(D & E)** are shown. The *x*-axis represents the repeat size interval starting from zero **(A & D)** or 5 **(B & E)** repeats, and *y*-axis defines the number of reads containing a repeat in the specified range. Peaks corresponding to an increased frequency between zero and 5 repeats are marked by a red arrow in the histogram **(A & D)**. To investigate the nature of these peaks, Sanger sequencing of the long-range PCR product, an aliquot of which was used in nanopore sequencing, was performed and the chromatograms for patients 553 **(C)** and 556 **(F)** are shown. Sanger sequencing chromatograms enabled only the allele with the smallest number of repeat units to be completely characterised, based on the position at which the sequence trace diverged. The chromatograms confirmed that samples 553 and 556 had alleles of 29 and 27 repeats respectively, and that the peaks between zero and 5 repeats **(A & D)** were absent from the Sanger sequencing trace and so represented artefacts of the STRique program that do not actually exist in the samples and were therefore removed from any subsequent analysis.

Supplementary Table 1. Genetic analysis of CTG18.1 locus in 119 FECD cases.

Sample ID	STR/TP-PCR genotype	Long-read nanopore genotype	Reads phased
550	11/31	11/29	YES
551	-/-	66/77	YES
552	11/11	10/10	NO
553	27/-	29/93	NO
555	11/24	12/23	YES
556	29/-	27/78	YES
557	11/11	11/11	NO
558	27/-	26/52	NO
574	11/17	10/16	YES
581	11/27	10/25	YES
582	17/-	17/70	YES
583	11/-	11/54	YES
585	11/-	12/66	YES
586	14/-	14/85	YES
587	11/11	11/11	NO
588	11/-	12/69	YES
589	11/27	11/25	YES
590	14/14	14/14	NO
592	-/-	63/75	NO
593	11/14	12/15	YES
594	15/-	15/107	YES
595	11/-	11/99	YES
596	17/-	17/98	YES
597	11/14	10/12	YES
598	11/11	12/12	NO
750	17/-	17/91	YES
751	17/-	17/75	YES
752	12/-	12/88	YES
753	11/-	11/52	YES
754	11/16	11/16	YES
755	15/17	16/16	YES
756	11/-	11/61	NO
757	14/-	15/85	YES
758	11/14	11/11	NO
760	11/-	12/73	YES
761	11/-	12/102	NO
762	11/-	11/90	YES

763	11/-	11/92	YES
765	14/17	14/14	YES
769	11/-	11/87	YES
773	14/-	14/91	YES
774	17/32	17/31	YES
775	11/-	11/87	YES
778	13/-	13/73	NO
779	-/-	78/91	YES
781	11/-	11/72	YES
785	11/-	11/81	YES
786	17/-	16/75	YES
790	13/-	13/71	NO
791	11/-	12/77	NO
802	11/-	12/80	YES
804	13/13	10/13	YES
807	11/11	11/11	NO
808	26/-	25/99	YES
810	26/-	26/90	NO
811	14/14	14/14	YES
813	27/-	27/50	NO
816	11/15	11/15	NO
817	14/24	14/23	YES
827	11/-	10/76	YES
831	11/14	11/11	NO
840	15/-	15/78	NO
842	17/-	16/88	YES
843	24/-	23/87	YES
844	14/-	11/67	NO
846	17/-	16/62	YES
848	11/17	10/16	YES
870	17/-	16/74	YES
871	11/-	11/69	YES
876	-/-	82/92	NO
877	11/-	10/91	YES
878	15/15	15/15	YES
885	16/-	16/103	YES
890	11/-	11/76	YES
894	14/-	14/71	YES
929	14/-	13/98	YES
931	21/-	20/103	YES
944	-/-	75/112	YES
946	27/-	26/118	YES

948	17/-	17/72	YES
949	-/-	62/78	NO
958	16/-	16/69	YES
965	17/32	16/31	YES
968	16/-	15/88	YES
991	11/26	10/23	YES
992	11/14	11/11	YES
1067	25/-	24/73	NO
1070	14/17	10/14	YES
1074	-/-	77/83	YES
1079	14/-	14/104	NO
1091	11/-	10/75	NO
1100	11/-	14/77	YES
1101	14/-	14/93	YES
1111	23/-	19/86	YES
1112	14/-	13/65	YES
1113	24/26	20/25	NO
1119	13/-	10/68	YES
1124	-/-	75/82	YES
1125	17/-	10/74	YES
1127	11/-	11/58	YES
1136	23/-	22/101	YES
1260	14/17	14/14	YES
1261	11/17	11/16	YES
1264	11/-	11/97	YES
1265	16/-	16/91	YES
1266	-/-	70/73	NO
1267	-/-	56/66	NO
1272	21/-	20/65	YES
1273	-/-	60/60	NO
1276	-/-	61/69	NO
1281	ND	14/79	YES
1282	ND	11/85	YES
1283	ND	11/68	YES
1284	ND	14/78	YES
1285	ND	11/84	YES
1286	ND	14/67	NO
1296	ND	14/79	YES
1297	ND	10/83	YES
1298	ND	14/66	YES

Sample ID is shown with the size of the repeat following STR/TP-PCR or nanopore sequencing and whether it was possible to phase the reads. -: size unknown. ND: No data.

Supplementary Table 2. Sequence quality metrics on base calling accuracy across the targeted region following nanopore sequencing.

Run (Number of samples)	Sample ID	Nanostat			
		Before filtering		After filtering	
		Total reads	Median Q score	Total reads	Median Q score
Flongle 1 (1)	802	92,941	12.4	37,886	13.7
Flongle 2 (9)	585	17,966	12.8	13,847	13.5
	586	3,841	12.7	2,818	13.5
	587	20,259	12.8	15,705	13.5
	588	37,194	12.6	25,858	13.5
	589	23,661	12.7	18,012	13.6
	590	22,500	12.4	15,092	13.4
	592	16,759	12.6	12,521	13.5
	593	4,429	13.0	3,566	13.6
	594	10,578	12.6	8,005	13.5
Flongle 3 (9)	791	1,454	13.3	1,023	13.9
	804	2,084	13.2	1,471	14.0
	807	2,092	13.2	1,453	13.9
	808	2,954	13.3	2,074	14.0
	810	2,766	13.1	1,798	13.9
	811	3,920	13.0	2,496	14.0
	813	3,314	13.1	2,030	14.0
	816	3,028	13.1	1,962	13.9
	817	7,645	13.4	5,599	14.0
Flongle 4 (10)	550	6,695	11.5	4,130	12.8
	551	7,811	11.4	4,828	12.8
	552	11,216	11.7	7,652	12.8
	555	7,978	11.8	5,535	12.8
	558	9,934	11.7	6,479	12.7
	574	18,516	11.4	10,854	12.7
	581	7,823	11.4	4,787	12.7
	582	8,951	11.4	5,391	12.7
	583	9,056	11.4	5,454	12.7
	947	6,646	11.5	3,984	12.7
Flongle 5 (10)	595	6,392	12.6	4,702	13.6
	596	6,543	12.7	4,987	13.6
	597	5,144	12.7	3,875	13.6
	598	6,564	12.6	4,862	13.5

	1281	7,112	12.6	5,272	13.6
	1282	6,801	12.7	5,224	13.6
	1283	6,543	12.6	4,937	13.6
	1284	8,396	12.7	6,277	13.6
	1285	9,327	12.6	7,035	13.6
	1286	6,652	12.6	4,966	13.7
Flongle 6 (13)	553	4,357	12.9	2,813	14.0
	690	10,017	13.7	7,179	14.3
	750	8,333	13.1	6,060	14.1
	751	12,662	12.9	8,139	14.1
	752	8,400	13.1	5,930	14.1
	753	21,076	13.1	14,466	14.1
	754	8,243	13.0	5,633	14.1
	755	31,487	13.0	21,165	14.1
	756	7,098	13.1	4,900	14.1
	757	5,260	13.2	3,702	14.1
	758	6,149	13.0	4,309	14.1
	762	5,099	13.5	2,321	14.1
	785	8,778	13.2	6,583	14.1
Flongle 7 (13)	760	9,038	13.0	6,701	13.8
	761	4,127	13.0	3,088	13.7
	763	19,985	13.0	14,913	13.8
	764	13,551	13.2	10,063	13.8
	765	27,229	13.1	20,801	13.7
	769	20,920	12.9	15,127	13.7
	773	14,333	13.1	11,338	13.7
	774	6,469	13.0	5,019	13.7
	775	6,664	13.1	4,859	13.7
	778	14,081	12.9	10,404	13.7
	779	15,319	13.0	11,343	13.7
	781	19,998	13.0	15,375	13.7
	786	23,661	13.0	14,400	13.8

Metrics for 7 flongle runs are presented. Nanostat was used to generate total read counts across the targeted region, before and after filtering to retain reads of 4,000 to 6,000 bases with a Q-score >10. Total reads and median Q-scores for each sample is shown. Note considerable per-sample read depth that was achieved for the assay that enabled high Q-score filtering, so that only high-quality reads were used for downstream analysis.

Supplementary Table 3. Genetic analysis of CTG18.1 locus in 83 non-FECD controls.

Sample ID	STR/TP-PCR genotype	Long-read nanopore genotype	Reads phased
1120	11/15	ND	ND
1138	14/14	ND	ND
1139	14/17	ND	ND
1140	11/14	ND	ND
1141	24/27	ND	ND
1142	11/17	ND	ND
1143	11/11	ND	ND
1144	11/11	ND	ND
1145	11/14	ND	ND
1146	15/25	ND	ND
1147	11/14	ND	ND
1148	11/11	ND	ND
1149	11/11	ND	ND
1150	11/11	ND	ND
1151	14/14	ND	ND
1152	14/30	ND	ND
1153	15/23	ND	ND
1154	11/24	ND	ND
1155	15/15	ND	ND
1156	17/23	ND	ND
1157	11/14	ND	ND
1158	11/29	ND	ND
1159	11/11	ND	ND
1160	17/17	ND	ND
1161	11/17	ND	ND
1162	27/27	ND	ND
1163	17/17	15/23	YES
1164	11/14	ND	ND
1165	11/11	ND	ND
1166	11/17	ND	ND
1167	14/17	ND	ND
1168	11/17	ND	ND
1169	11/11	ND	ND
1201	11/14	ND	ND
1202	11/17	ND	ND
1203	11/26	ND	ND

1204	17/-	15/80	YES
1205	11/23	ND	ND
1206	15/15	10/17	YES
1207	11/17	ND	ND
1208	17/24	ND	ND
1209	11/14	ND	ND
1210	11/11	10/15	NO
1211	14/23	ND	ND
1212	14/24	ND	ND
1213	14/14	ND	ND
1214	14/26	ND	ND
1215	11/13	ND	ND
1216	14/27	ND	ND
1217	11/17	ND	ND
1218	11/11	ND	ND
1219	11/22	ND	ND
1220	19/31	ND	ND
1221	11/23	ND	ND
1222	14/17	ND	ND
1223	11/29	ND	ND
1224	14/14	ND	ND
1225	14/14	ND	ND
1226	15/28	ND	ND
1227	14/15	ND	ND
1228	14/17	ND	ND
1229	11/14	ND	ND
1230	17/22	ND	ND
1231	11/17	ND	ND
1232	11/31	ND	ND
1233	15/25	ND	ND
1234	10/11	ND	ND
1235	11/11	ND	ND
1236	11/26	ND	ND
1237	14/14	ND	ND
1238	11/14	ND	ND
1239	11/11	ND	ND
1240	11/17	ND	ND
1241	14/17	ND	ND
1242	17/17	ND	ND
1243	17/27	ND	ND
1244	11/11	ND	ND
1245	11/29	ND	ND

1246	17/17	ND	ND
1247	11/35	ND	ND
1249	11/15	ND	ND
1250	11/26	ND	ND
1251	11/17	ND	ND

Sample ID is shown with the size of the repeat following STR/TP-PCR or nanopore sequencing and whether it was possible to phase the reads. -: size unknown. ND: No data.
